# Factors Associated with Frailty According to Gender of Older Adults Living Alone

**DOI:** 10.3390/healthcare9040475

**Published:** 2021-04-16

**Authors:** Hye-Young Jang, Ji-Hye Kim

**Affiliations:** 1 College of Nursing, Hanyang University, Seoul 04763, Korea; white0108@hanyang.ac.kr; 2 College of Nursing, Woosuk University, Chonbuk 55338, Korea

**Keywords:** frailty, living alone, gender, older adults

## Abstract

This study was conducted to identify the factors associated with frailty according to gender of older adults living alone in Korea. Data from the National Survey of the Living Conditions of Korean Elderly in 2017 were used. Participants were 2340 older adults who live alone. Frailty was determined based on the frailty criteria developed by van Kan et al. that consist of fatigue, resistance, ambulation, and illness. The collected data were analyzed using descriptive statistics, chi-squared test, *t*-test, Jonckheere–Terpstra test and multinomial logistic regression. Among the older men living alone, 47.7% were in the pre-frail and 5.1% were in the frail. On the other hand, 51.8% were in the pre-frail and 12.2% were in the frail among the older women living alone. The factors associated with frailty according to gender are as follows. In males, depressive symptoms, limitation in IADL, and number of medications in pre-frail; BMI, limitation in IADL, and number of chronic diseases in frail. In females, depressive symptoms, number of chronic diseases, age, and nutritional status in pre-frail; limitation in IADL, depressive symptoms, age, number of chronic diseases, number of medications, nutritional status in frail. Based on the findings of this study, it is considered necessary to approach frailty management considering gender as well as the classification of frailty.

## 1. Introduction

In Korea, as the rapid aging and changes in traditional values about family, living alone among older adults are increasing year after year. According to a 2019 older adult statistics report published by Statistics Korea, the percentage of older adults aged 65 or more who live alone is 34.2%, and the number of older adults living alone is expected to increase rapidly in the future [1].

In addition to the physiological vulnerability caused by aging, older adults who live alone are often vulnerable in terms of physical and psychosocial aspects [2,3]. Compared to those who live with their spouse or children, older adults who live alone are not only less educated and are at a lower economic status but have a higher prevalence of chronic diseases, multi-morbidity, and depressive symptoms [2]. Further, they show poor nutritional status and a markedly higher percentage of safety incidents, falls, and abuse [2]. As shown here, older adults living alone are vulnerable in terms of the physical, mental, and social aspects of health, underlining the need for societal attention and support to help them maintain multilateral aspects of health and function as well as independence.

On the other hand, as the importance of maintaining the function and preventing disability among older adults is emphasized in order to maintain their quality of life and minimize the societal cost of older adult caregiving, interest in the frailty among older adults is increasing. Frailty refers to a clinical syndrome, distinguished from normal aging that is characterized by a state of vulnerability with poor homeostatic resolution of exposure to stressors as a result of accumulated age-related defects in various physiological systems [4,5]. The incidence of frailty among community-dwelling older adults ranges from 4.9% to 27.3% worldwide and is 7.8% in Korea [6]. Frailty can be viewed as a stage preceding impairment or disease that has been associated with adverse outcomes, such as death, hospitalization, loss of activities of daily living (ADL), physical restriction, falls, and fractures [7]. Therefore, the prevention and management of frailty in older adults is crucial to prevent impairments and long-term care in the older adult population.

The most widely used operational definition and criterion for frailty is the “phenotype frailty” by Fried et al. [5]. It consists of unintentional weight loss, exhaustion, slow walking speed, weak grip strength, and low physical activity as the frailty phenotype. It is performance-based measures of physical function including slow walking speed and grip strength, and thus well-trained investigators and extra measuring devices were required [5]. In comparison, the FRAIL scale (fatigue, resistance, ambulation, illnesses, and loss of weight) consists of five simple questions without any objective measurement. Thus it is economical, easy and quick to use, and is useful for frailty screening [8]. Each item of the FRAIL scale is related to the biological index of the weakness, and clinical validity has been reported as it shows predictive power for health outcomes similar to that of other frailty scales [9,10].

Frailty has a continuous, dynamic of change leading from normal aging to pre-frailty, frailty, and geriatric illness, and is reversible according to active efforts [4,11,12]. In a systematic review and meta-analysis of longitudinal studies on the progression of frailty using frailty phenotype, Kojima et al. [13] found that 3.3% and 40.3% of frail older adults reverted to a non-frailty stage and pre-frailty stage, respectively [13]. As shown here, frailty in older adults is a reversible process, which calls for early intervention to delay progression to pre-frailty or frailty and reverse frailty to pre-frailty or non-frailty. Particularly, when the degree of frailty is deemed as continuous, the pre-frailty stage is a crucial period in which the signs of frailty can be reversed or prevented and thus is a critical stage for preventive interventions [5,14].

In this context, identifying the predictors of frailty is essential to maximize the effects of interventions to prevent or improve frailty. According to a previous study on the predictors of frailty in older adults, frailty in older adults was associated with sociodemographic characteristics [15,16,17,18,19], health-related factors [5,11,15,17,18,19,20,21,22], mental and cognitive factors [17,18,20,23], and social factors [11,15,20,24], showing that multilateral approaches are needed in interventions for the prevention and improvement of frailty. However, as most studies have involved the older adult population in the United States and Europe, it is inappropriate to apply these findings directly to the Korean older adult population. There are cultural differences, such as socioeconomic situations, psychological and social health aspects, medical systems for the elderly, and perceptions of elderly caregiving [9,25,26] so it is necessary to identify the factors affecting frailty in the context of Korean culture. Furthermore, because most studies have focused on the frailty stage in older adults, identifying the predictors of pre-frailty is crucial considering the significance of the pre-frailty stage.

Thus, in this study, we examine the differences of frailty by gender among older adults living alone (Aim 1) and try to identify the different factors influencing pre-frailty and frailty on a continuous line (Aim 2).

### 1.1. Study Design

This study is a secondary descriptive survey investigating the factors associated with frailty by classification of frailty in Korean older adults living alone using the National Survey of the Living Conditions of Korean Elderly (NSLCKE) in 2017.

### 1.2. Data and Ethical Considerations

**Figure 1:**
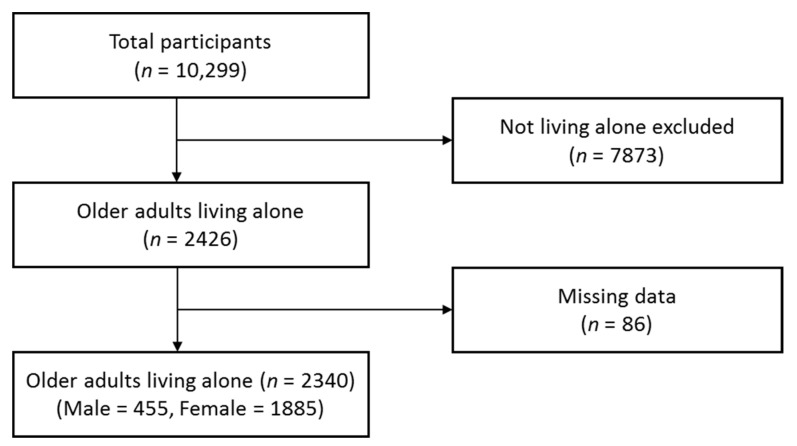
Flow chart of study.

In this study, we obtained the data of the NSLCKE in 2017 according to the Korea Institute for Health and Social Affairs (KIHASA) policy for disclosure and management of raw data. This study was approved by the Institutional Review Board of W-University to which the researcher was affiliated (No. WS-2021-1). Raw data were collected from 12 June to 28 August 2017, and a written informed consent was obtained from all participants of the survey prior to data collection. The target population of the NSLCKE was older adults aged 65 years or older who live in communities in 17 cities in Korea and provinces nationwide, and a total of 10,299 people participated in the survey. In this study, data for older adults who live alone were taken from the entire 10,299 participants of the survey. After excluding those with missing values in variables related to frailty (*n* = 86), we analyzed data from 2340 participants (Figure 1).

### 1.3. Measurements

To identify the factors associated with frailty in Korean older adults living alone from a multidimensional perspective, we consist of sociodemographic factors, health-related factors, mental and cognitive factors, and social factors as the independent variables with reference to previous studies.

#### 1.3.1. Sociodemographic Characteristics

As sociodemographic characteristics, age, education level, and total household income were included. Age was divided into 65–74 years, 75 years or over, and education level was measured as total years of education. Total household income was measured as total annual income.

#### 1.3.2. Health-Related Factors

Health-related factors included number of chronic diseases, history of falls, BMI, nutritional status, and number of medications. Number of chronic diseases refers to sum of the number of comorbidities diagnosed by a physician and included; hypertension, stroke, hyperlipidemia, coronary heart disease, diabetes, thyroid disease, arthrosis, osteoporosis, depression, incontinence and chronic obstructive pulmonary disease, etc. History of falls refers to falls (fall, slip, or collapse) that occurred in the past year, and the responses were categorized as yes or no. BMI was classified into underweight (BMI < 18.5), normal (18.5 ≤ BMI ≤ 22.9), overweight (23 ≤ BMI ≤ 24.9), and obesity (BMI ≥ 25) based on the WHO Asia-Pacific criterion [27]. Nutritional status was measured using the Korean version of the Determine Your Nutritional Health Checklist base on the Nutrition Screening Initiative [28,29]. The checklist includes 10 items which measures nutritional management status; food restriction due to an illness, two meals or fewer a day, insufficient dairy intake, insufficient food expense, dining alone, changes in weight, and difficulty with meal preparation. Each item is given a score of 1–4 points according to the weight, so the range of possible total scores is 0–21 points, and a higher score indicates poorer nutritional status. Number of medications refers to sum of the number of physician-prescribed medications taken daily for three months or longer.

#### 1.3.3. Mental and Cognitive Factors

Mental and cognitive factors included depressive symptoms and cognitive function. Depressive symptoms were measured using the Geriatric Depression Scale Short Form-Korean version (GDSSF-K) [30,31]. The GDSSF-K consist of 15 items using a binary (0 or 1) scale. The total score ranges from 0 to 15, with higher scores indicating higher level of depression. The cutoff score for depressive symptoms was set to 8 [31]. In the present study, we classified the scores into depressive symptoms (≥8) and no depressive symptoms (≤7). Cognitive function was measured using the Korean version of the Mini-Mental State Examination for Dementia Screening (MMSE-DS). The MMSE-DS consist of 30 items using a binary (0 or 1) scale. The total score ranges from 0 to 30, with higher scores indicating higher cognitive function.

#### 1.3.4. Social Factors

Social factors included social activity, limitation in IADL, and use of healthcare services. The social activity was measured based on the frequency of participation for each types of activities (learning, clubs, social groups, political groups, volunteering, religion), in which no participation was given 0 point, less than once a month was given 1 point, once a month was given 2 points, once in two weeks was given 3 points, more than once a week was given 4 points. The total score ranged from 0 to 24 points, and a higher score indicates more active social participation. Limitation in IADL was measured using the 10-item Korean Instrumental Activities of Daily Living (K-IADL), which was originally developed by Lawton and Brody [32] and adapted into Korean by Won et al. [33]. With reference to a previous study [34], we defined “no limitation” as being able to perform all 10 items and “limitation” as inability to perform any one of the 10 items. Use of healthcare services was classified into yes or no based on the utilization of a healthcare facility in the past month.

#### 1.3.5. Frailty

Frailty was measured using the FRAIL scale. The FRAIL scale consists of 5 items such as fatigue, resistance, ambulation, illness, and loss of weight. The scale is based on a maximum score of 5, and the scores are interpreted as non-frail (0), pre-frail (1–2), and frail (3–5) [8]. In this study, we used the 4 items (fatigue, resistance, ambulation, and illness) of the FRAIL scale because the NSLCKE not include information about weight loss. Fatigue was measured using the item “Have you recently been much less active or feeling less motivated to be active?” (yes = 1 no = 0). Resistance was measured using the item “Do you have difficulty climbing 10 steps without rest?” (not difficult at all = 0, slightly difficult = 0, very difficult = 1, cannot do at all = 1). Ambulation was measured using the item “Do you have difficulty walking 400 m without an assistive device?” (not difficult at all = 0, slightly difficult = 0, very difficult = 1, cannot do at all = 1). Illness was measured using the item “Have you been diagnosed with a chronic disease by a physician?” (0–4 sum of the comorbidities = 0, ≥5 sum of the comorbidities = 1). We interpreted the scores as non-frail (0), pre-frail (1–2), and frail (3–4).

### 1.4. Data Analysis

Data were analyzed using SPSS/WIN 22.0 software (SPSS, Chicago, IL, USA). The variables were analyzed with descriptive statistics, and the differences in variables by gender were analyzed with X^2^ test and *t*-test. The differences in variables by classification of frailty (non-frail/pre-frail/frail) were analyzed with ordinal X^2^ test (age, history of falls, BMI, depressive symptoms, limitation in IADL, use of health services), Jonckheere–Terpstra test (education, household income, number of chronic diseases, nutritional status, number of medications, cognitive function, social activity), and post-hoc analyzed using a Bonferroni correction (*p*-value < 0.017 for significance). To identify the predictor of frailty by classification of frailty, we performed multinomial logistic regression for “non-frail/pre-frail” and “pre-frail/frail. To explore the aim of this study, the researchers determined that the multinomial logistic regression model was more suitable than the ordinal logistic regression model, based on previous studies showing different characteristics according to frailty status [19,35,36]. All *p* values < 0.05 were considered to indicate statistical significance.

## 2. Results

### 2.1. General Characteristics of Participants by Gender

Participant demographic characteristics are shown in Table 1. Except for depressive symptoms, all measured variables for frailty had a statistically significant difference in gender.

### 2.2. Differences in Classification of Frailty by Gender

The participants were divided into three groups (non-frail, pre-frail, frail) to analyze by classification of frailty (Table 2). The male groups significantly differed in all measured variables for frailty. Except for BMI, all measured variables had a statistically significant difference in the female groups.

### 2.3. Factors Associated with Frailty by Classification of Frailty by Gender

Multinomial logistic regression was performed for each group to identify the predictors of frailty by classification of frailty; between non-frail and pre-frail, and between pre-frail and frail. All variables confirmed to be significant in the univariate analysis were entered in the logistic regression (Table 3).

The predictors of frailty between non-frail and pre-frail in male were number of medications, depressive symptoms, and limitation in IADL. The predictors of frailty between pre-frail and frail were number of chronic diseases, BMI, and limitation in IADL.

The predictors of frailty between non-frail and pre-frail in female were age, number of chronic diseases, nutritional status, and depressive symptoms. The predictors of frailty between pre-frail and frail were age, number of chronic diseases, nutritional status, number of medications, depressive symptoms, and limitation in IADL.

## 3. Discussion

In response to the growing number of older adults living alone along with the burgeoning of the older adult population, it is essential to develop effective interventions that prevent frailty and help maintain maximum health function in older adults living alone. Thus, this study analyzed the data of the NSLCKE in 2017 to identify the predictors of frailty by classification of frailty in Korean older adults living alone.

Among the older men living alone, 47.7% were in the pre-frailty and 5.1% were in the frailty, while 51.8% and 12.2% were among the older women living alone, respectively, indicating that the elderly women were more vulnerable. The results are consistent with a study by Op et Veld et al. [15], which shows that women are two times more vulnerable than men in the elderly among Dutch, and in the English Longitude Study of Ageing (ELSA), women were 1.28 times more likely to be vulnerable than men [16]. It is thought that the higher the risk of frailty for the elderly women living alone is related to the higher proportion of elderly women over 75 years of age and the poor physical health of the older women compared to the older men [37]. This is supported by the study, which shows that the health of older women is more vulnerable in the number of chronic diseases, the fall rate, and the number of medications taken. It is also confirmed that there were differences in sex in muscle mass, physical activity, fat ratio, hormones among the older with frailty, and it can be assumed that the older man have protective factors in the development and progression of frailty [37,38,39]. In addition, women tended to be weaker than men, but mortality rates of older men were higher, and the frailty became worse over time [37,40]. Therefore, further research is needed to understand the perception of differences in frailty status by gender and the pathways that influence them.

However, the data used in this study did not have any known questions about weight loss, so it is important to note the interpretation of the results because we used the fatigue, resistance, ambulation, and illness criteria of the FRAIL scale. Researchers in this study judged that it is reasonable to evaluate frailty on a four criteria because the fatigue items among the FRAIL could be used to assess the biological factor of older and the weight loss criteria were found to be less likely to differ in the prevalence of frailty [39]. In addition, it should be noted that the evaluation of frailty through self-reported questions can overestimate one’s condition, so that the prevalence of frailty is higher than frailty phenotypes based objective measurements [41]. According to a systematic review of studies that utilized a modified version of the frailty phenotype criteria by Fried et al. [5] using the Survey of Health, Ageing, & Retirement in Europe (SHARE), physical activity and weight loss criteria were most frequently modified, and the classification and predictability for frailty differed depending on which items were modified, how items were modified, how they were measured, and how missing values were processed [39]. Therefore, a standardized method is needed to measure each frailty criterion, and studies should be conducted to examine the frailty using the same scale with validity and reliability.

In this study, depressive symptoms were the most important predictor of pre-frail in older men and women living alone, which is consistent with the results of a previous study on older adults aged 75 years or older and socially disadvantaged older adults [18,23]. Depressive symptoms diminish interest in daily living and level of activity, and mental and psychological factors such as depressive symptoms increase the risk of frailty by affecting health even among older adults who are capable of independent living [42]. In a recent study that compared the health status of older adults living alone by sex, the rate of loneliness, depressive symptoms, suicidal ideation, and suicide attempts was higher among older men living alone than their female counterparts. These factors were identified as important determinants of quality of life in older men [43]. As shown here, living alone may exacerbate mental health problems such as depressive symptoms and loneliness in older men, which is not only an important risk factor for physical frailty but also a factor that threatens quality of life, necessitating effective depression interventions and gender-specific programs for older adults who live alone. Moreover, the impact of depression on frailty was greater among those aged 84 years or younger compared to those aged 85 years or older [23]; therefore, age must be considered in developing interventions for frailty prevention.

In this study, older adults with limitation in IADL were found to be the predictor of pre-frail and frail in older men living alone and frail in older women living alone and this is contextually in line with the results that the level of limitation in IADL differs among the non-frailty, pre-frailty, and frailty groups and that limitation in IADL is a predictor of the progression from non-frailty to pre-frailty [11,15]. A diminished ability to carry on with IADL and reduced independence can contribute to depressive symptoms as well as frailty in older adults [34], and depressive symptoms in turn affects frailty [18,23], calling for appropriate exercise programs to maintain and strengthen physical function in older adults who live alone.

In this study, to identify the impact of the nutritional aspect on frailty, we used BMI and nutritional status as the variables. The results showed that the underweight older adult predicted the frailty in older men living alone and the nutritional status predicted the pre-frailty and frailty in older women living alone, as found in previous studies [11,15,18]. However, in contrast to our results, a few recent studies reported that abdominal obesity, as opposed to underweight, can predict progression to frailty [16,20]. As an increase of fat is more associated with diminished functional performance than is reduced muscle mass in older adults [44], and most older adults have sarcopenic obesity characterized by reduced muscle mass with increased body fat even when they have normal or increased body weight [45], interventions should be focused on strengthening muscles instead of simply losing or gaining weight in order to lower the risk of progression to frailty. Moreover, reduced muscle strength and weight loss are considered as major markers of physical frailty [5], so further studies on the relationship between frailty and nutrition in older adults should not only measure BMI but also measure lean body mass and muscle mass and quality to identify on their relationship with frailty. In addition, older adults who live alone lack motivation to prepare and consume meals and also have poor nutrition and diet due to financial reasons [46]. Social support such as continuous nutrition education, meal services, and home meal deliveries are needed to improve their quantitative and qualitative nutritional status.

In this study, the number of chronic diseases was identified as a predictor of frail in older men living alone and pre-frail and frail in older women living alone, as found in previous studies [11,15,18]. According to the NSLCKE in 2017, older adults in one-person households were diagnosed with an average of 3.2 chronic diseases by a physician, showing a higher prevalence of chronic disease and multiple comorbidities compared to older adults in other types of living arrangements [2]. In particular, chronic diseases, such as diabetes mellitus, chronic obstructive pulmonary disease, cancer, and heart failure, were identified as the predictors of progression and improvement of frailty in older men [11]. Taken together, we can predict that vulnerability to frailty increases with increasing number of chronic diseases and that certain diseases can further elevate the risk of frailty. Therefore, monitoring and support are needed to help older adults living alone to continuously manage their chronic diseases and cope with them.

The number of medications taken was shown to be a predictor of pre-frail in older men and frail of the older women living alone, which is consistent with previous results [20,21,22]. The number of medications taken daily is directly linked to the number of comorbidities, so it may be an objective indicator of health status [22]. Older adults may be prescribed more medications to manage various chronic diseases that develop as they age. In particular, among various types of medications, the use of medications for managing cardiovascular risk was identified as a predictor of frailty [5,22]. Polypharmacy may induce or worsen frailty by having an adverse impact on the factors that influence frailty and the markers included in the definition of frailty [21,47]. As older adults are subject to changes in drug interactions and drug–disease interactions as a result of pharmacokinetic and pharmacodynamic changes, they may experience frequent and serious adverse drug reactions. Adverse drug reactions may directly or indirectly induce frailty by having a negative impact on physical frailty or worsening the state of frailty [48]. Prescribing additional medications without identifying such adverse drug interactions may further exacerbate the conditions, so the appropriateness of each drug therapy for older adults on polypharmacy should be assessed. Thus, pharmacists and healthcare professionals as well as physicians providing care for older adults in the community must plan and manage individualized drug therapy for older adults.

Finally, age has been identified as a predictor in predicting the pre-frail and frail in older women living alone, which supports the results of a previous studies that revealed the strong positive relationship between age and frailty [15,16,17,18]. The difference in the degree of frailty by age is thought to be due to the change and the difference in multidimensional health and demographic factors along with the increase in age. Therefore, a differentiated and comprehensive approach according to age is important to maintain physical, mental and social well-being for the elderly living alone.

As described above, there were different predictor for each classification of frailty and gender in older living alone, and this can be understood in the same context as that in a previous study where the risk factors and protective factors associated with stepwise progression or improvement differed in the frailty pathway [11]. Based on the findings of this study, it is considered necessary to approach frailty management considering gender as well as the classification of frailty because the risk factors vary depending on gender and stage of frailty. For example, for the older men living alone, the psychosocial approach centered on the management of depression is the first priority for prevention of progression from non-frailty to pre-frailty and the construction of a nutrition management system aimed at maintaining proper weight should be given priority for prevention of progression from pre-frailty to frailty.

This study has a few limitations. Because the NSLCKE in 2017 did not contain information about weight loss, we excluded the “loss of weight” criterion from the five frailty criteria proposed by van Kan et al. [8] and only used the remaining four criteria. As frailty classification and predictability were confirmed to vary depending on the number of frailty markers and types of variations [39,41], further studies should use standardized methods to measure the five criteria in their examination of classification and predictors of frailty in older adults living alone. In addition, because this study is a cross-sectional design, attention must be taken when interpreting the causal relationship between predictors and frailty. Depression, for example, may be the cause of frailty, but on the contrary, depression may appear as a result of frailty. In future studies, studies should utilize longitudinal data in order to identify the causative relationship between frailty and its predictors. Despite these limitations, this study is significant in that it identified the predictors by classification of frailty in Korean older adults living alone using a nationally representative dataset, which could contribute to designing interventions to prevent and delay frailty and ultimately enhance the quality of life and dignity of older adults living alone.

### Implications for Nurses

As the number of older adult living alone increases, nurses need to be concerned about the frailty of them. The development of gender-based tailored program to the classification of frailty to prevent frailty of older adults living alone should be considered. In addition, we suggest that nurses use the FRAIL scale, which is an economical and easy-to-use scale for screening frailty.

## 4. Conclusions

In this study, we analyzed data from the NSLCKE to identify the predictors of frailty by classification in older adults living alone. The results showed that the predictors of frailty varied by classification of frailty among older adults living alone. In males, depressive symptoms, limitation in IADL, and number of medications in pre-frail; BMI, limitation in IADL, and number of chronic diseases in frail. In females, depressive symptoms, number of chronic diseases, age, and nutritional status in pre-frail; limitation in IADL, depressive symptoms, age, number of chronic diseases, number of medications, nutritional status in frail. Based on the findings of this study, it is considered necessary to approach frailty management considering gender as well as the classification of frailty.

In the future, studies should use longitudinal data to examine the causative relationship between frailty and its predictors. Furthermore, based on our results, customized frailty prevention interventions that are tailored to the specific classification of frailty and gender should be developed and implemented.

## Figures and Tables

**Table 1:** General Characteristics of Participants by Gender. *n* = 2340.

Variables	Categories	Total (*n* = 2340)	Male (*n* = 455)	Female (*n* = 1885)	X^2^ or t (*p*)
		*n* (%) or Mean ± SD	*n* (%) or Mean ± SD	*n* (%) or Mean ± SD	
Age (year)		75.71 ± 6.77	74.06 ± 6.98	76.11 ± 6.65	33.80 (<0.001)
	65~74	1091 (46.6)	268 (58.9)	823 (43.6)	
	≥75	1251 (53.4)	187 (41.1)	1063 (56.4)	
Education (year)		5.57 ± 4.64	8.61 ± 4.57	4.84 ± 4.35	16.44 (<0.001)
Household income (10,000 won/year)		1168.43 ± 937.65	1329.05 ± 1279.30	1129.64 ± 830.26	3.17 (0.002)
Number of chronic diseases		3.18 ± 1.89	2.55 ± 1.77	3.34 ± 1.88	−8.15 (<0.001)
Experiences of falls	Yes	459 (11.1)	56 (12.3)	425 (22.5)	23.53 (<0.001)
	No	3685 (88.9)	399 (87.7)	1460 (77.5)	
BMI (kg/m^2^)	Underweight	98 (4.2)	29 (6.4)	69 (3.7)	42.78 (<0.001)
	Normal weight	958 (40.9)	204 (44.7)	754 (40.0)	
	Overweight	593 (25.3)	142 (31.1)	451 (23.9)	
	Obesity	693 (29.6)	80 (17.8)	612 (32.4)	
Nutritional status	(range: 0–21)	5.05 ± 3.23	5.37 ± 3.43	4.97 ± 3.17	2.30 (0.022)
Number of medications		4.41 ± 3.32	4.01 ± 3.60	4.50 ± 3.24	−2.64 (0.009)
Depressive symptoms	(range: 1–15)	5.15 ± 4.42	5.13 ± 4.44	5.16 ± 4.41	0.09 (0.759)
	Yes	701 (30.0)	139 (30.5)	562 (29.8)	
	No	1639 (70.0)	316 (69.5)	1323 (70.2)	
Cognitive impairment	(range: 0–30)	24.35 ± 4.08	25.75 ± 3.44	24.01 ± 4.15	9.27 (<0.001)
Social activity	(range: 0–24)	3.17 ± 3.14	2.25 ± 2.88	3.39 ± 3.17	−7.44 (<0.001)
Limitation in IADL	Yes	600 (14.5)	97 (20.9)	656 (34.8)	30.53 (<0.001)
	No	3545 (85.5)	358 (78.7)	1229 (65.2)	
Use of health services	Yes	1916 (81.9)	321 (70.5)	1595 (84.6)	48.88 (<0.001)
	No	424 (18.1)	134 (29.5)	290 (15.4)	
Frailty	Non-frail	894 (38.2)	215 (47.3)	679 (36.0)	30.62 (<0.001)
	Pre-frail	1193 (51.0)	217 (47.7)	976 (51.8)	
	Frail	253 (10.8)	23 (5.1)	230 (12.2)	

BMI, body mass index; IADL, instrumental activities of daily living. SD, standard deviation. BMI category descriptions: less than 18.5 kg/m^2^, underweight; 18.5 to 22.9 kg/m^2^, normal weight; 23 to 24.9 kg/m^2^, overweight; 25 kg/m^2^ or more, obesity.

**Table 2:** The Differences in Frailty related Variables by Gender. *n* = 2340.

Variables	Categories	Male (*n* = 455)			X^2^ or J-T (*p*)	Female (*n* = 1885)			X^2^ or J-T (*p*)
		Non-Frail ^a^ (*n* = 215)	Pre-Frail ^b^ (*n* = 217)	Frail ^c^ (*n* = 23)		Non-Frail ^a^ (*n* = 679)	Pre-Frail ^b^ (*n* = 976)	Frail ^c^ (*n* = 230)	
		*n* (%) or Mean ± SD				*n* (%) or Mean ± SD			
Age (year)	65~74	136 (63.3)	122 (56.2)	10 (41.7)	4.89 (0.027)	381 (56.1)	388 (39.8)	54 (23.5)	86.69 (<0.001)
	≥ 75	79 (18.3)	95 (43.8)	14 (58.3)		298 (43.9)	588 (60.2)	176 (76.5)	
Education (years) *		9.21 ± 4.72	8.03 ± 4.33	8.44 ± 4.64	−1.97 (0.049) a > b	5.94 ± 4.57	4.48 ± 4.19	3.09 ± 3.43	−9.01 (<0.001) a > b > c
Household income (10,000 won/year) *		1474.30 ± 1544.01	1215.88 ± 996.01	1040.08 ± 650.56	−3.20 (0.001) a > b	1293.03 ± 941.28	1053.48 ± 775.17	970.68 ± 600.51	−8.86 (<0.001) a > b > c
Number of chronic diseases *		1.91 ± 1.28	2.91 ± 1.85	5.03 ± 1.66	7.99 (<0.001) a < b < c	2.20 ± 1.25	3.71 ± 1.78	5.09 ± 1.90	23.32 (<0.001) a < b < c
History of falls	Yes	19 (8.8)	32 (14.7)	4 (17.4)	4.03 (0.045)	98 (14.4)	235 (24.1)	92 (39.8)	64.56 (<0.001)
	No	196 (91.2)	185 (85.3)	19 (82.6)		581 (85.6)	741 (75.9)	139 (60.2)	
BMI (kg/m^2^)	Underweight	10 (4.7)	10 (4.6)	8 (24.8)	3.93 (0.048)	15 (2.5)	38 (3.9)	16 (7.0)	0.05 (0.829)
	Normal weight	100 (46.7)	93 (43.1)	10 (43.5)		280 (41.2)	390 (40.0)	84 (36.5)	
	Overweight	62 (29.0)	76 (35.2)	3 (13.0)		181 (26.7)	221 (22.7)	48 (20.9)	
	Obesity	42 (19.6)	37 (17.1)	2 (8.7)		203 (29.9)	326 (33.4)	82 (35.7)	
Nutritional status *		4.12 ± 2.66	6.28 ± 3.58	8.52 ± 3.85	8.14 (<0.001) a < b < c	3.46 ± 2.21	5.45 ± 3.18	7.38 ± 3.40	18.35 (<0.001) a < b < c
Number of medications *		2.74 ± 2.63	4.86 ± 3.80	7.86 ± 4.50	8.17 (<0.001) a < b < c	2.95 ± 2.45	4.94 ± 3.08	7.22 ± 3.55	19.17 (<0.001) a < b < c
Depressive symptoms	Yes	10 (4.7)	114 (52.5)	16 (66.7)	123.25 (<0.001)	35 (5.2)	375 (38.4)	153 (66.5)	378.18 (<0.001)
	No	205 (95.3)	103 (47.5)	8 (33.3)		644 (94.8)	602 (61.6)	77 (33.5)	
Cognitive function *		26.46 ± 3.00	25.18 ± 3.63	24.52 ± 4.09	−4.09 (<0.001) a > b,c	25.02 ± 3.69	23.80 ± 4.14	21.93 ± 4.54	−9.58 (<0.001) a > b > c
Social activity *		2.82 ± 3.29	1.83 ± 2.38	1.01 ± 1.85	−4.10 (<0.001) a > b,c	4.12 ± 3.36	3.16 ± 3.06	2.24 ± 2.46	−8.640 (<0.001) a > b > c
Limitation in IADL	Yes	26 (12.1)	55 (25.3)	16 (69.6)	36.26 (<0.001)	128 (18.9)	358 (36.7)	171 (74.0)	216.97 (<0.001)
	No	189 (87.9)	162 (74.7)	7 (30.4)		551 (81.1)	618 (63.3)	60 (26.0)	
Use of health services	Yes	137 (63.4)	163 (75.1)	22 (95.7)	13.85 (<0.001)	540 (79.5)	853 (87.4)	203 (88.3)	17.67 (<0.001)
	No	79 (36.6)	54 (24.9)	1 (4.3)		139 (20.5)	123 (12.6)	27 (11.7)	

J-T, Jonckheere-Terpstra test; ^a^, non-frail; ^b^, pre-frail; ^c^, frail; * significant difference among groups based on Bonferroni correction post-hoc test.

**Table 3:** Multinomial Logistic Regression of Frailty Status by Gender.

Variables	Categories	Male (*n* = 455)		Female (*n* = 1885)	
		Non-Frail vs. Pre-Frail (*n* = 432)	Pre-Frail vs. Frail (*n* = 240)	Non-Frail vs. Pre-Frail (*n* = 1655)	Pre-Frail vs. Frail (*n* = 1206)
		OR (95% CI)	OR (95% CI)	OR (95% CI)	OR (95% CI)
Age (year)	65~74	1 (referent)	1 (referent)	1 (referent)	1 (referent)
	≥75	1.611 (0.973–2.667)	1.045 (0.272–4.017)	1.491 (1.158–1.919) **	1.601 (1.078–2.379) *
Education (years)		1.048 (0.982–1.118)	1.048 (0.919–1.195)	1.004 (0.970–1.039)	0.997 (0.948–1.048)
Household income (10,000 won/year)		1.000 (1.000–1.000)	1.000 (0.999–1.001)	1.000 (1.000–1.000)	1.000 (1.000–1.000)
Number of chronic diseases		1.135 (0.912–1.414)	1.661 (1.157–2.384) **	1.751 (1.577–1.944) ***	1.327 (1.191–1.477) ***
History of falls	Yes	0.939 (0.441–1.996)	2.465 (0.585–10.376)	1.213 (0.882–1.668)	1.336 (0.939–1.901)
	No	1 (referent)	1 (referent)	1 (referent)	1 (referent)
BMI (kg/m^2^)	Underweight	1.190 (0.380–3.729)	14.082 (1.641–120.801) *	1.168 (0.564–2.421)	0.814 (0.371–1.786)
	Normal weight	1.073 (0.541–2.130)	3.322 (0.512–21.539)	0.934 (0.702–1.242)	0.700 (0.472–1.039)
	Overweight	1.750 (0.857–3.573)	0.972 (0.116–8.161)	0.806 (0.587–1.107)	0.830 (0.530–1.301)
	Obesity	1 (referent)	1 (referent)	1 (referent)	1 (referent)
Nutritional status		1.021 (0.930–1.120)	0.998 (0.845–1.179)	1.112 (1.058–1.170) ***	1.067 (1.011–1.125) *
Number of medications		1.117 (1.004–1.243) *	0.960 (0.804–1.148)	1.036 (0.981–1.094)	1.108 (1.046–1.173) ***
Depressive symptoms	Yes	18.962 (8.812–40.803) ***	1.457 (0.411–5.164)	7.319 (1.941–10.843) ***	1.872 (1.315–2.665) ***
	No	1 (referent)	1 (referent)	1 (referent)	1 (referent)
Cognitive function		0.923 (0.846–1.007)	0.969 (0.826–1.137)	0.993 (0.955–1.031)	0.963 (0.920–1.008)
Social activity		0.942 (0.861–1.032)	0.859 (0.661–1.117)	0.977 (0.938–1.017)	0.951 (0.890–1.016)
Limitation in IADL	Yes	1.979 (1.003–3.791) *	5.319 (1.523–18.576) **	1.294 (0.956–1.752)	2.738 (1.875–3.998) ***
	No	1 (referent)	1 (referent)	1 (referent)	1 (referent)
Use of health services	Yes	1.102 (0.621–1.956)	5.572 (0.728–42.631)	0.758 (0.534–1.075)	0.680 (0.407–1.137)
	No	1 (referent)	1 (referent)	1 (referent)	1 (referent)

* *p* < 0.05, ** *p* < 0.01, *** *p* < 0.001.

## Data Availability

The data that support the findings of this study are available from the corresponding author, upon reasonable request.
